# Análise Computacional da Dinâmica dos Fluídos na Substituição da Valva Aórtica Transcateter

**DOI:** 10.36660/abc.20201002

**Published:** 2020-10-13

**Affiliations:** 1 Universidade de São Paulo Faculdade de Medicina Instituto do Coração (InCor) São PauloSP Brasil Instituto do Coração (InCor), Faculdade de Medicina da Universidade de São Paulo (FMUSP), São Paulo, SP - Brasil

**Keywords:** Mecânica de Fluídos, Substituição da Valva Aórtica Transcateter/métodos, Hemodinâmica, Fluxo Sanguíneo Regional

A substituição da válvula aórtica transcateter (TAVR), uma cirurgia cardíaca minimamente invasiva, foi introduzida por Cribier et al.,^1^ como alternativa à cirurgia cardíaca tradicional a céu aberto no tratamento de indivíduos com estenose valvar aórtica importante e de alto risco cirúrgico, devido à idade avançada ou à presença de múltiplas comorbidades.^2^ Após os primeiros esforços pioneiros, o advento de válvulas protéticas inovadoras e abordagens e dispositivos mais refinados tecnologicamente, o uso de TAVR para pacientes com risco cirúrgico intermediário tem sido uma tendência mundial.^3^ No entanto, a variação no posicionamento e orientação da válvula protética após o procedimento de TAVR pode produzir alterações significativas na hemodinâmica aórtica e nas tensões correspondentes na parede do vaso.^4^

Na aorta, existem duas categorias de tensões na parede do vaso. A primeira categoria de tensão é o resultado do atrito entre o sangue em movimento e a parede do vaso, que é proporcional à velocidade do sangue, afastando-se da camada íntima da parede do vaso. Esse tipo de tensão é conhecido como tensão de cisalhamento na parede aórtica (*wall shear stress* — WSS). A segunda categoria de tensão se deve à variação na pressão de pulso gerada durante o ciclo cardíaco. Nesta categoria, existem tensões circunferenciais, axiais e radiais transferidas para todas as camadas da parede do vaso. Com o avançar da idade, a aorta aumenta de tamanho, o arco muda sua forma de um semicírculo quase perfeito e o vaso geralmente se torna mais tortuoso.^5^ Além disso, a mudança na curvatura natural da aorta introduz a dinâmica de fluxo secundária e a assimetria no fluxo, que influenciam diretamente a distribuição e magnitude da tensão de cisalhamento sobre a parede do vaso.

Dentre as modalidades de imagem disponíveis, a tomografia computadorizada (TC) é amplamente considerada o método padrão-ouro para estudo e análise da aorta, artérias coronárias e femorais. Avanços recentes usando um detector de ampla cobertura (256 ou 320 cortes) e TC de fonte dupla de alta frequência tornaram possível usar menos contraste e uma dose de radiação menor. Embora a TC possa representar as complexidades geométricas e funcionais da aorta, atualmente ela se limita a capturar uma imagem instantânea do fluxo sanguíneo em um determinado instante durante o ciclo cardíaco.

Por outro lado, a ressonância magnética (RM) de fluxo quadridimensional (4D) é uma nova técnica que avalia o fluxo sanguíneo aórtico no espaço tridimensional em função do tempo, o que permite a quantificação da hemodinâmica aórtica.^6^ Esta nova técnica de aquisição de imagens pode melhorar nossa compreensão acerca da dinamicidade inerente do fluxo sanguíneo aórtico. No entanto, a TC pode ser melhorada com o modelagem em fluidodinâmica computacional (*computational fluid dynamics* — CFD), que pode estimar parâmetros hemodinâmicos anteriormente incomensuráveis para se entender o comportamento biomecânico do fluxo sanguíneo em vasos normais e doentes.

Na ausência de um meio prontamente aplicável para medir diretamente a WSS, a modelagem em CFD tem sido aplicada em imagens de TC e RM para se compreender os padrões espaciais e temporais da WSS e a influência da fluidodinâmica aórtica nesse parâmetro.^7^^-^^9^ Usando imagens de TC como entrada em uma modelagem em CFD, Celis et al.,^10^ demonstraram que pequenas variações no ângulo de inclinação da válvula aórtica podem modificar a natureza do fluxo e produzir alterações na distribuição da WSS na parede aórtica.

A CFD é um método viável que tem sido usado há anos^11^ para determinar o fluxo de fluidos e o modelo 3D das artérias coronárias e pode simular um fluxo preciso nos vasos com base em um conjunto de parâmetros. Para fluidos incompressíveis, a maioria das análises da CFD resolve o conjunto de equações de continuidade e de Navier-Stokes que governam o movimento dos fluidos. Esse conjunto de equações inclui equações diferenciais parciais não lineares baseadas no princípio da conservação da massa e do momento. A equação de Navier-Stokes descreve o movimento viscoso dos fluidos^12^ e, de acordo com a lei da viscosidade de Newton, a relação entre a tensão de cisalhamento e a taxa de cisalhamento de um fluido, sujeito a tensão mecânica, é uma constante para uma dada temperatura e pressão, sendo definida como a viscosidade ou coeficiente de viscosidade. Fisiologicamente, isso significa que o fluxo sanguíneo no sistema cardiovascular é igual à variação da pressão arterial dividida pela resistência do sistema.^13^

Apesar da disponibilidade de poderosos pacotes de software de CFD utilizados para modelar o fluxo dos fluidos, como o ANSYS FLUENT, o OpenFOAM, o SIMVascular e o ADINA,^14^ os métodos em CFD atuais têm um grande custo de tempo computacional, o que os impede de serem usados em grandes coortes de pacientes. Esse custo de tempo advém basicamente da complexidade dos modelos, que precisam da geometria anatômica do paciente, propriedades teciduais, condições de carga hemodinâmica e seleção adequada das técnicas de modelagem. Uma possível solução de mudança de paradigma para os gargalos nos métodos em CFD atuais é incorporar algoritmos de aprendizado de máquina (*machine learning* — ML)^15^ para agilizar a análise computacional, começando com a modelagem geométrica até a configuração do modelo computacional e a conclusão da simulação.

Liang et al.,^16^ desenvolveram recentemente uma nova abordagem de aprendizado de máquina que demonstrou a viabilidade do uso de ML como um substituto rápido e preciso da CFD para estimar os campos hemodinâmicos em estado estacionário da aorta torácica humana. Em sua abordagem, a CFD é tratada como uma caixa preta e o algoritmo de ML aprende a relação não linear entre os dados de entrada e saída da CFD. Em média, o método proposto levou minutos para executar uma simulação de CFD para cada modelo de aorta, o que parece ser rápido o suficiente para aplicações clínicas.

As medições in vivo da hemodinâmica e das tensões correspondentes na aorta não são práticas. Portanto, a modelagem em CFD é amplamente usada para estimar esses parâmetros, mas é demorada e cara do ponto de vista computacional. Os modelos de ML podem ser uma alternativa promissora para simulações de CFD para auxiliar nas decisões clínicas e no tratamento com base em pacientes específicos. Isso pode levar a melhores resultados clínicos em muitos estudos, como a identificação da melhor posição e orientação da válvula protética no procedimento de TAVR.
