# Supplementary material for: Análise Computacional da Dinâmica dos Fluídos na Substituição da Valva Aórtica Transcateter
Source: Arq Bras Cardiol. 2020 Oct 13;115(4):688–9. [Article in Portuguese] doi: 10.36660/abc.20201002 (PMC8386972; doi:10.36660/abc.20201002)
Supplement: Translation

# Translation: Computational Analysis of Fluid Dynamics in the Transcatheter Aortic Valve Replacement

**Affiliations:** 1 Sao PauloSP Brazil Heart Institute (InCor), University of Sao Paulo Medical School (FMUSP), Sao Paulo, SP - Brazil

**Keywords:** Flow Mechanics, Transcatheter Aortic Valve Replacement/methods, Hemodynamics, Regional Flood Flow

Transcatheter aortic valve replacement (TAVR), a minimally invasive heart surgery, was introduced by Cribier et al.^1^ as an alternative to the traditional open-heart surgery in the treatment of individuals with severe aortic valve stenosis and at high surgical risk due to advanced age or the presence of multiple comorbidities.^2^ After the first pioneering efforts, the advent of innovative prosthetic valves, and more technologically refined approaches and devices, the use of TAVR for patients with intermediate surgical risk has been a worldwide trend.^3^ However, variation in the prosthetic valve positioning and orientation post TAVR procedure can produce significant changes in the aortic hemodynamics and the corresponding stresses in the vessel wall.^4^

Within the aorta, there are two categories of vessel wall stress. The first category of stress is the result of the friction between the moving blood and the vessel wall, which is proportional to the blood speed, moving away from the intimate layer of the vessel wall. This kind of stress is known as wall shear stress (WSS). The second category of stress is due to the variation in pulse pressure generated during the cardiac cycle. In this category, there are circumferential, axial and radial stress transferred to all vessel wall layers. With advancing age, the aorta enlarges, the arch changes shape from a near-perfect semicircle, and the vessel generally becomes more tortuous.^5^ Moreover, the change in the natural curvature of the aorta introduces secondary flow dynamics and flow asymmetry, which directly influence WSS distribution and magnitude over the vessel wall.

Among the available imaging modalities, computed tomography (CT) is widely considered the gold standard method for studying and analyzing the aorta, coronary and femoral arteries. Recent developments using a wide coverage detector design (256 or 320 slices) or high-frequency dual-source CT have made it possible to use less contrast and a lower radiation dose. Although CT can present the geometrical and functional complexities of the aorta, it is currently limited to capture a snapshot of the blood flow at a defined instant of time during the cardiac cycle.

On the other hand, four-dimensional (4D) flow magnetic resonance imaging (MRI) is a novel technique with the capability of assessing aortic blood flow in three-dimensional space as a function of time, which permits the quantification of aortic hemodynamics.^6^ This new imaging acquisition technique may improve our understanding of the inherent dynamicity of aortic blood flow. However, CT can be improved with computational fluid dynamics (CFD) modeling, which can compute previously unmeasurable hemodynamic parameters to understand the biomechanical behavior of blood flow in both normal and diseased vessels.

In the absence of a readily applicable means to directly measure WSS, CFD has been applied in CT and MRI images to understand both the spatial and temporal patterns of WSS and the influence of aortic flow dynamics on this parameter.^7^^–^^9^ Using CT images as the input of a CFD model, Celis et al.^10^ demonstrated that small variations of the aortic valve tilt angle could modify the nature of the flow and produce changes in the distribution of the WSS over the aorta wall.

CFD is a feasible method that has been used for ages^11^ in determining fluid flow and 3D model of coronary arteries and can simulate an accurate vessel flow based on a set of given parameters. For incompressible fluids, most CFD analysis solve the Navier-Stokes and continuity equations that govern fluid motion. This set of equations includes non-linear and partial differential equations based on the principle of conservation of mass and momentum. Navier-Stokes equation describes the viscous motion of fluids^12^ and, according to Newton’s law of viscosity, the relationship between the shear stress and shear rate of a fluid, subjected to mechanical stress, is a constant for a given temperature and pressure, and is defined as the viscosity or coefficient of viscosity. Physiologically, this means that the blood flow in the cardiovascular system is equal to the change of blood pressure divided by the system resistance.^13^

Despite the availability of powerful CFD software packages to model fluid flow, such as ANSYS FLUENT, OpenFOAM, SIMVascular, and ADINA,^14^ the current CFD methods have large computational time cost, which prevents them from being used in large patient cohorts. This time cost basically comes from the complexity of the models, which need patient anatomic geometries, tissue properties, hemodynamics loading conditions, and proper selection of modeling techniques. A potential paradigm-changing solution to the bottlenecks in current CFD methods is to incorporate machine learning (ML) algorithms^15^ to expedite computational analysis, starting from geometry modeling to computational model setup, and simulation completion.

Liang et al.^16^ have recently developed a novel machine learning approach that demonstrated the feasibility of using ML as a fast and accurate surrogate of CFD to estimate steady-state hemodynamic fields of human thoracic aorta. In their approach, CFD is treated as a black box, and the ML algorithm learns the nonlinear relationship between CFD input and output. On average, the proposed method took minutes to run a CFD simulation for each aorta model, which seems to be fast enough for clinical applications.

In vivo measurements of parameters hemodynamics and the corresponding stress in the aorta are not practical. Therefore, CFD is widely used to estimate these parameters, but it is time consuming and computationally expensive. ML models can be a promising alternative for CFD simulations to aid clinical decisions and treatment based on specific patients. This can lead to better clinical results in many studies, such as the identification of the best position and orientation of the prosthetic valve in the TAVR procedure.
